# Association between tendency to overeat and the incidence of prediabetes: Aichi Workers’ Cohort Study

**DOI:** 10.1265/ehpm.26-00084

**Published:** 2026-09-10

**Authors:** Shalini Enon Perera Paththamesthrige, Masaaki Matsunaga, Zean Song, Midori Takada, Baruck Tegegn Endale, Nanami Nishio, Yuna Hattori, Tahmina Akter, Mohammad Hassan Hamrah, Hanson Gabriel Nuamah, Chisato Fukuda, Shuang Wang, Atsuhiko Ota, Rei Otsuka, Koji Tamakoshi, Hiroshi Yatsuya

**Affiliations:** 1 Department of Public Health and Health Systems, Nagoya University Graduate School of Medicine, Nagoya, Aichi, Japan; 2 Department of Public Health, Fujita Health University School of Medicine, Toyoake, Aichi, Japan; 3 Department of Epidemiology of Aging, Research Institute, National Center for Geriatrics and Gerontology, Obu, Aichi, Japan; 4 Department of Nursing, Nagoya University School of Health Sciences, Nagoya, Aichi, Japan

**Keywords:** Overeating, Eating behavior, Prediabetes, Diabetes, Cohort study

## Abstract

**Background and aims:**

Evidence regarding the association between the tendency to overeat and prediabetes or diabetes is still limited. Therefore, this study aimed to investigate the association between the tendency to overeat and the incidence of prediabetes among young and middle-aged Japanese working population.

**Methods:**

The baseline survey was conducted among 2886 local government workers, aged 20–65 in central Japan in 2013. Participants were asked, “Do you tend to overeat?” and self-reported as either “Yes” or “No” in the survey. Prediabetes was defined as fasting blood glucose ≥100 mg/dL, or participants starting diabetes medication or having reported a new history of diabetes until 2018. Cox model was used to obtain hazard ratios (HRs) and 95% confidence intervals (CIs) adjusted for socio-demographic, lifestyle and baseline metabolic factors.

**Results:**

During a mean follow-up period of 4.2 years, 541 prediabetes incidences were reported. The tendency to overeat, compared to not overeating, was associated with a significantly higher incidence of prediabetes (HR: 1.33, 95% CI: 1.09–1.62). This association was similar in both sexes but was attenuated in females (male: HR; 1.32; 95% CI, 1.06–1.64; females: HR 1.38; 95% CI, 0.86–2.23) in model 2.

**Conclusion:**

Tendency to overeat was significantly associated with the increased incidence of prediabetes in young and middle-aged Japanese workers. Therefore, targeted screening for this might contribute to effective prevention strategies for reducing incidence of prediabetes among this population.

**Supplementary information:**

The online version contains supplementary material available at https://doi.org/10.1265/ehpm.26-00084.

## Graphical Abstract

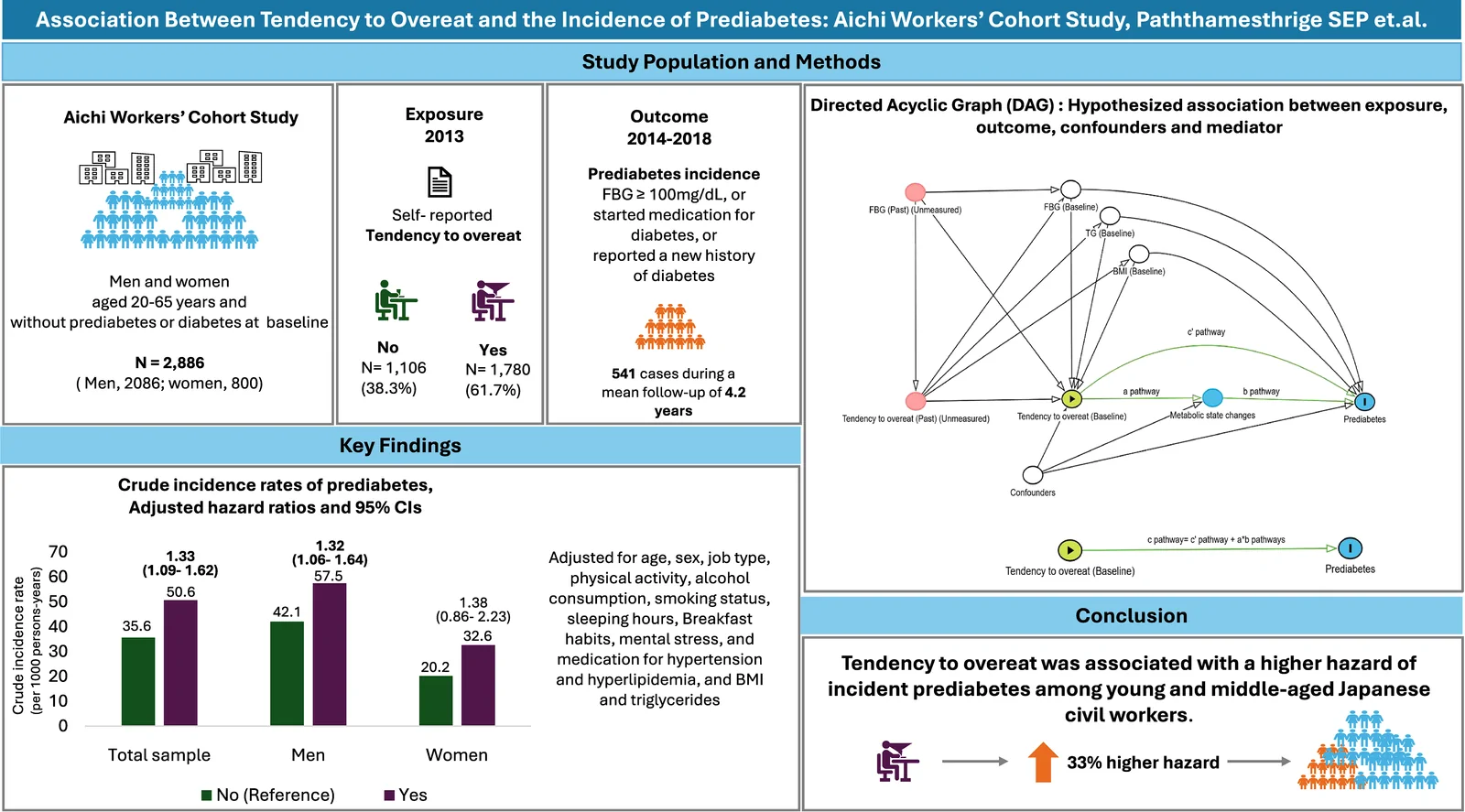

## Introduction

The global prevalence of diabetes and prediabetes has been gradually increasing, with a particularly high burden in Asia [1]. In Japan and Southeast Asia, the age-standardized prevalence of impaired fasting glucose (IFG) and impaired glucose tolerance (IGT) among adults is notably high [2]. In a Japanese population, one study found that individuals with prediabetes had a substantially increased risk of progression to diabetes within five years [3]. These findings draw attention to identifying and addressing risk factors that may contribute to prediabetes in the Japanese general population.

Prediabetes is a condition of abnormal glucose homeostasis, distinguished by elevated blood glucose levels that surpass the normal range but do not meet the criteria for diabetes [4, 5] as well as its macrovascular and microvascular complications [6].

The tendency to overeat is a measure of dietary behavior that is characterized by overconsumption of food or eating beyond satiety [7]. Unlike a clinical entity of binge eating disorder, which is defined as recurrent, psychologically distressing and hard-to-control episodes of consuming large amounts of food with loss of control [8], overeating is a broader behavior tendency that is not necessarily pathological. However, overeating was associated with the five-year development of adiposity in adolescents [7]. It was reported that relatively small daily excess energy intake, when sustained overtime, may cumulatively lead to weight gain and increased cardiometabolic risk [9] highlighting the importance of sustained healthy eating habits, including avoidance of habitual overeating, for metabolic diseases prevention. Another longitudinal study found that overeating was associated with the development of metabolic syndrome even after accounting for the potential confounders [10]. While experimental studies in both animals and humans have also demonstrated short-term [11, 12] and long-term [12, 13] overeating can induce insulin resistance and glycemic dysregulation, few populations based cross-sectional studies reported the effect of overeating on glycemic dysregulation [14, 15]. Nevertheless, the association between tendency to overeat and prediabetes has not been thoroughly investigated in population-based studies.

Building on these findings, and recognizing overeating as a modifiable dietary behavior, this study hypothesizes that the tendency to overeat is associated with prediabetes incidence among young and middle-aged Japanese local government workers.

## Methods

### Study population

The Aichi Workers’ cohort study is an ongoing epidemiological study on the investigation of non-communicable diseases. The study recruited local government workers in central Japan. Approximately every five years, a self-administered questionnaire is distributed to participants. The current study is based on data from 2013, which was the fourth wave of the survey. The study protocol received ethical approval from the Ethics Review Committee of Nagoya University School of Medicine, Nagoya, Japan (Approval number: 2013-0005).

### Inclusion criteria

Participants who provided informed written consent for us to obtain their worksite annual health check-up data at baseline and follow-up and provided complete medical histories and lifestyles through the self-administered questionnaire at baseline were included in the current study (n = 3721).

### Exclusion criteria

**Fig. 1:**
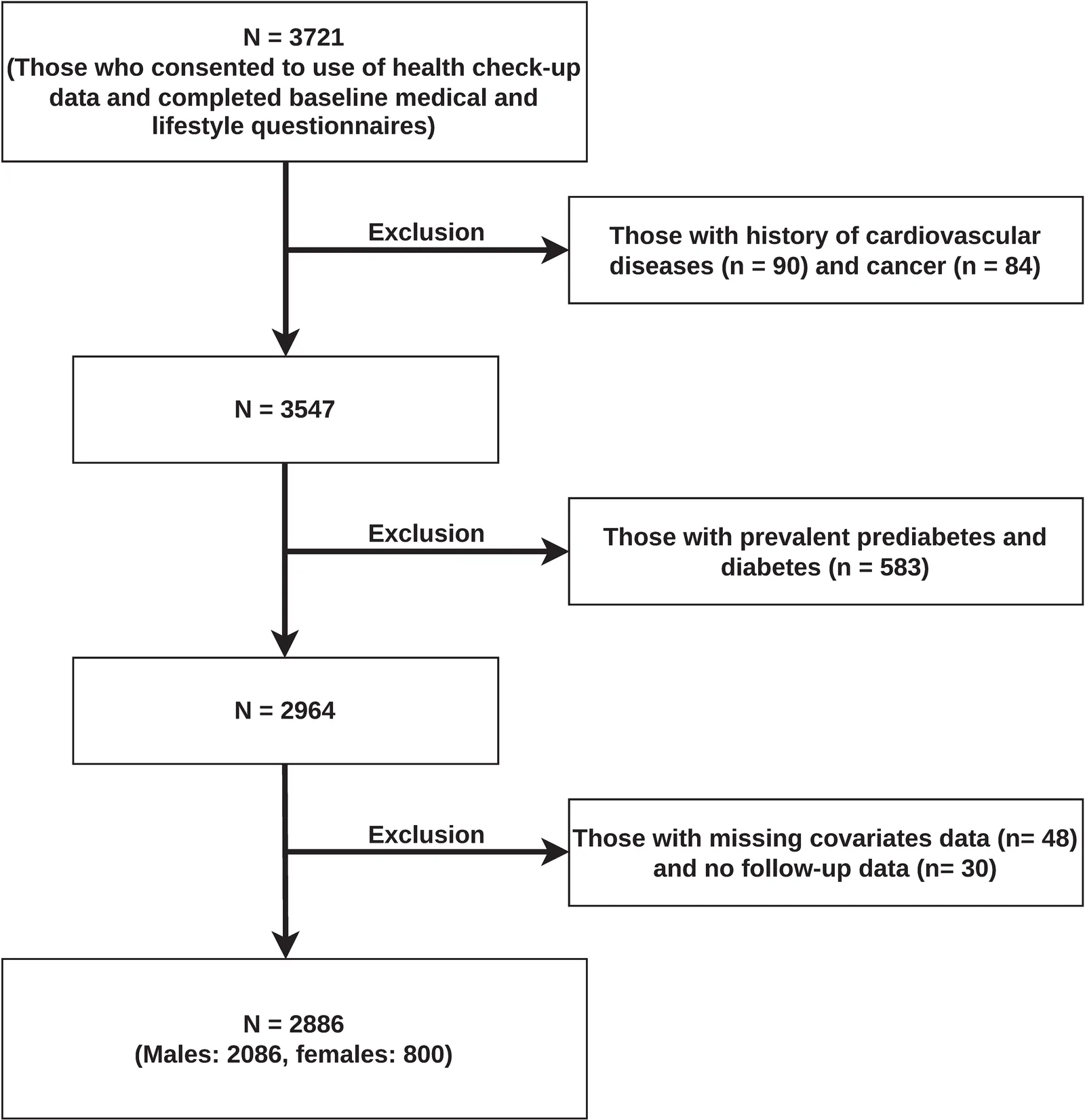
Flow chart of participants selection.

Of total 3,721 participants, those with a history of cardiovascular diseases (CVD) (n = 90), and cancer (n = 84) were excluded. Additionally, 583 participants with prediabetes including diabetes mellitus at baseline were excluded. Of the 2,964 remaining participants, 48 were excluded due to missing covariates data and 30 participants due to lack of at least one-year follow-up data. Finally, 2,886 (male: 2086, female: 800) participants were included in the analysis (Fig. 1).

### Measures

#### Tendency to overeat

Overeat was self-reported by the participants in the questionnaire through a single question asked about the previous year: “Do you tend to overeat?” participants answered either “yes” or “no.”

#### FBG measurement

Blood for FBG measurement was collected at the annual worksite health check-up. After overnight fasting for ≥8 hours, blood was drawn from the participants and serum glucose levels were measured using the hexokinase method. FBG was measured annually from 2013 to 2018 as part of routine worksite health examinations.

#### Endpoint

In the current study, prediabetes including diabetes mellitus were defined as FBG level ≥100 mg/dL [16], or participants who started medication for diabetes mellitus, or having reported a new history of diabetes mellitus until 2018. Diabetes mellitus and other medical conditions were self-reported. Participants who reported diabetes mellitus were asked to provide consent for the researchers to confirm the history with their physicians.

#### Follow-up

Participants were followed from the baseline year (2013) until the date of censoring or prediabetes incidence. Participants were censored upon loss to follow-up or at the end of the follow-up year in 2018. Proportion of loss to follow-up is 16.5% in the current study.

#### Assessment of covariates

Height and weight were measured with light clothes and without shoes in the morning at the fasting status. Measurements were taken to the nearest 0.1 cm and 0.1 kg respectively. Weight was divided by the square of height in meters to obtain the body mass index (BMI). Waist circumference (WC) was measured at the height of umbilicus, to the nearest 0.1 cm. Longitudinal changes of BMI were calculated by subtracting the baseline values from those measured at the prediabetes incidence or censoring year or end of follow-up. Physical activity performed in a day was calculated by multiplying the metabolic equivalent task (MET) value for each relevant activity by the duration of the activity in hours and summing up the METs·hours/day for all activities and used as a continuous variable. Smoking status was categorized into four categories including never smokers, past smokers, current smokers with <20 cigarettes per day, and current smokers with ≥20 cigarettes per day. The amount of alcohol consumption per day was calculated as ethanol intake per day (g/day). Sleeping hours were categorized into three as, ≤5 hours, 6–7 hours, ≥8 hours. Breakfast habits were categorized as not every day and every day. Participants self-reported their average daily food intake frequency for the last year using a semi-quantitative food frequency questionnaire and presented their total energy intake as kilocalorie per day. Energy-adjusted carbohydrate intake was calculated by dividing carbohydrate-derived energy (kcal/day) by total energy intake (kcal/day) and expressed as a percentage. Similarly, energy-adjusted fat intake was calculated by dividing fat-derived energy (kcal/day) by total energy intake (kcal/day) and expressed as a percentage. Job type was categorized as clerical and professional or technical into two. Medication for hypertension and hyperlipidemia were self-reported as “yes” and “no”. Mental stress was assessed using the 4-items Perceived Stress Scale (PSS-4) and was treated as a continuous variable. Blood triglycerides (TG) were measured, at the mandatory annual health check-up at the worksite after overnight fasting for ≥8 hours. TG was analyzed by the hexokinase method.

#### Directed Acyclic Graph

The Directed Acyclic Graph (DAG) illustrates the hypothesized relationships between an exposure, and the outcome while taking consideration of confounders and mediator. In the present study, confounders represent a set of the following variables: age, sex, job type, physical activity, alcohol consumption, smoking status, sleeping hours, breakfast habit, mental stress and medications for hypertension and hyperlipidemia. Another set of confounding variables was indicated as baseline metabolic state which includes BMI, TG and FBG. Mediator is expressed as metabolic state change, which is a variable indicating longitudinal changes of BMI from 2013 to prediabetes incidence, censoring or end of the follow-up (Supplementary Fig. 1). Multicollinearity among covariates was assessed using variance inflation factors (VIFs). In addition, an alternative DAG based on a different causal framework was constructed to investigate the association (Supplementary Fig. 2).

### Statistical analysis

For descriptive purposes, TG were log-transformed to approximate a normal distribution and presented as geometric means with 95% confidence intervals in the baseline characteristics table. However, they were analyzed in their original scale in subsequent statistical analyses. Differences between tendency to overeat and not overeating were tested using an independent t-test for continuous variables and a chi-square test for categorical variables.

A Cox-regression hazard model was used to estimate the risk of prediabetes by calculating the hazard ratios (HRs) and 95% confidence intervals (CIs). Multivariable models were constructed according to the DAG. Namely, Model 1 included age, sex, job type, physical activity, alcohol consumption, smoking status, sleeping hours, breakfast habit, mental stress and medications for hypertension and hyperlipidemia. Model 2 included variables in Model 1 and variables indicating baseline metabolic state: BMI (kg/m^2^), TG (mg/dL). No evidence of problematic multicollinearity was observed among the covariates included in the Cox-regression hazard model, with all VIF values below 5. In addition, based on the alternative casual framework, a supplementary Cox regression hazard model was constructed including age, sex, job type, physical activity, alcohol consumption, smoking status, sleeping hours, breakfast habit, and mental stress (Supplementary Method 3).

Mediation analysis was conducted including longitudinal changes of BMI to Model 2 using PROCESS macro for SPSS (Version 5.0). The direct, indirect, and total effects of tendency to overeat on prediabetes incidence were estimated (B, regression coefficient), and lower and upper limits of the 95% bootstrap confidence intervals (CIs) were obtained using 5,000 bootstrap samples.

Additional analyses stratified by sex and smoking status (never, past, current) were conducted in Model 2. Stratified analysis by smoking status was done only in men as the small number of female participants with smoking history. Multiplicative interactions of overeat categories with sex as well as smoking status categories were also examined.

Sensitivity analyses were performed by censoring participants who developed diabetes mellitus (FBG ≥126 mg/dL [16], new medication used for diabetes mellitus, self-reported history of diabetes mellitus during follow-up). A different sensitivity analysis was conducted by excluding those who developed diabetes mellitus during follow-up from the analysis. All statistical analyses were performed using SPSS version 29. All statistical tests were two-sided, and p values <0.05 were considered statistically significant.

## Results

### Baseline characteristics

The study included 2,886 participants, comprising 2,086 males and 800 females. Among the participants, 1,106 were classified as not tending to overeat, while 1,780 were classified as tending to overeat. Participants with a tendency to overeat were younger (mean age: 46.1 ± 6.3), and more likely to be past smokers (22.7%), had elevated FBG and TG levels at baseline. BMI, WC, prevalence of hyperlipidemia, total energy intake, energy-adjusted fat intake, the daily amount of physical activity, the proportion of participants who skipped breakfast, and the proportion of those with sleep duration ≤5 hours were also higher in participants with tendency to overeat compared to those who did not (Table 1).

**Table 1:** Baseline characteristics based on tendency to overeat, Aichi Workers’ Cohort Study, n = 2886.

		**Tendency to overeat**		**p**

		**No** **N = 1,106**	**Yes** **N = 1,780**	
Age (years)		47.5 (6.4)	46.1 (6.3)	<0.001
Male		790 (71.4)	1296 (72.8)	0.41
Body mass index (kg/m^2^)		21.3 (2.5)	23.3 (3.1)	<0.001
Waist circumference (cm)		76.0 (7.6)	80.9 (8.8)	<0.001
Job type	Clerical	491 (44.4)	822 (46.2)	0.35
	Professional/Technical	615 (55.6)	958 (53.8)	
Mental stress (PSS-4)		7.2 (2.4)	7.8 (2.7)	<0.001
Alcohol consumption (g/day)		16.2 (22.2)	14.3 (20.9)	0.02
Smoking status	Never	738 (66.7)	1165 (65.4)	0.005
	Past	201 (18.2)	404 (22.7)	
	<19 cigarettes/day	95 (8.6)	128 (7.2)	
	≥20 cigarettes/day	72 (6.5)	83 (4.7)	
Sleeping hours	≤5 hours	129 (11.7)	292 (16.4)	<0.001
	6–7 hours	561 (50.7)	964 (54.2)	
	≥8 hours	416 (37.6)	524 (29.4)	
Breakfast habit	Not everyday	172 (15.6)	335 (18.8)	0.03
	Everyday	934 (84.4)	1,445 (81.2)	
Total energy intake (Kcal/day)		1,956 (639.5)	2,151 (770.6)	<0.001
Energy-adjusted carbohydrate intake (%)		54.61(8.50)	54.80(8.13)	0.56
Energy-adjusted fat intake (%)		24.92 (6.54)	25.80 (6.56)	<0.001
Physical activity (METs*h/day)		36.6 (3.2)	37.0 (3.6)	0.006
Medication for hypertension		70 (6.3)	131 (7.4)	0.30
Medication for dyslipidemia		36 (3.3)	94 (5.3)	0.01
Blood triglycerides (mg/dL)		80.6 (78.3–83.1)	89.1 (86.5–91.0)	<0.001
Fasting blood glucose (mg/dL)		88.7 (5.6)	89.3 (5.6)	0.006

METs indicate metabolic equivalent tasks; PSS-4, Perceived stress score-4 item. Data with the unit of measurement displayed on table were presented as mean (standard deviations) for continuous variables and the number (percentage) for categorical variables. Differences in continuous variables were tested using an independent t-test, and categorical variables were tested using a chi-square test. Blood triglycerides were log-transformed and tested, and the geometric mean and the 95% confidence interval are presented.

### Prediabetes incidence risk related to tendency to overeat

During a mean follow-up time of 4.2 years, 541 individuals developed prediabetes. The crude incidence rate of prediabetes was 50.6 per 1,000 person-years for participants with the tendency to overeat, while 35.6 per 1,000 person-years for participants without the tendency to overeat. In Model 1, participants with the tendency to overeat had 52% higher hazard ratio for prediabetes (HR: 1.56, 95% CI: 1.29–1.88). In Model 2, this association remained statistically significant (HR: 1.33, 95% CI: 1.09–1.62) (Table 2). The supplementary Cox regression hazard analysis based on the alternative DAG yielded similar findings (Supplementary Table 6).

**Table 2:** Tendency to overeat and prediabetes: Cox regression, Aichi Workers’ Cohort Study, n = 2886.

	**Tendency to overeat**	

	**No**	**Yes**
**Total participants** (n/N)	166/1,106	375/1,780
Crude incidence rate (/1000 person-years)	35.6	50.6
Model 1	1 (Ref)	1.56 (1.29–1.88)
Model 2	1 (Ref)	1.33 (1.09–1.62)
**Male participants** (n/N)	138/790	306/1,296
Crude incidence rate (/1000 person-years)	42.1	57.5
Model 1	1 (Ref)	1.52 (1.23–1.87)
Model 2	1 (Ref)	1.32 (1.06–1.64)
**Female participants** (n/N)	28/316	69/484
Crude incidence rate (/1000 person-years)	20.2	32.6
Model 1	1 (Ref)	1.69 (1.07–2.65)
Model 2	1 (Ref)	1.38 (0.86–2.23)

n indicates the number of incident prediabetes; N, the number of participants; Ref, reference

Note: Model 1 includes age, sex, job type, physical activity, alcohol consumption, smoking status, sleeping hours, breakfast habit, mental stress and medications for hypertension and hyperlipidemia. In the sex-stratified analyses, sex was not included as a covariate. Model 2 includes Model 1 and body mass index, triglycerides.

P for interaction between tendency to overeat categories and sex categories are 0.69.

### Mediation analysis

**Fig. 2:**
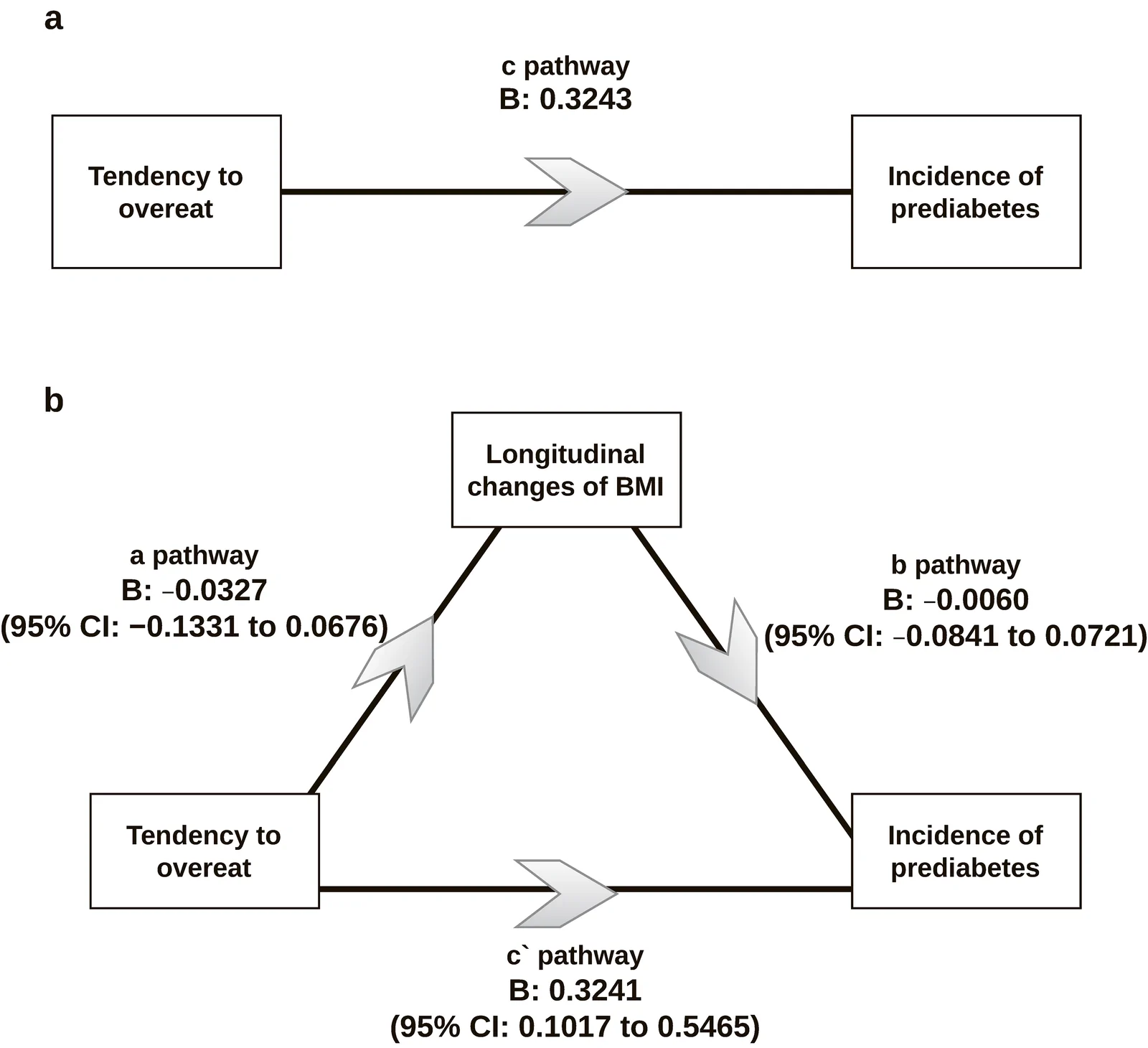
Mediation analysis of tendency to overeat, BMI change, and incidence of prediabetes. Panel a-. The total effect (c pathway) of the tendency to overeat (independent variable; X) on the incidence of prediabetes (dependent variable; Y). Panel b- The indirect effect of the tendency to overeat (independent variable; X) on the incidence of prediabetes (dependent variable; Y) through the longitudinal changes of BMI (mediator; M). a pathway is the effect of X on M. b pathway is effect of M on Y. The mediated or indirect effect is a*b pathways. c` pathway is the direct effect of X on Y. B, regression coefficient, 95% CI, lower and upper limits of the 95% bootstrap confidence interval.

Mediation analysis showed that longitudinal changes of BMI did not mediate the association between tendency to overeat and prediabetes incidence (Fig. 2). The estimated direct effect was (B: 0.3241, 95% CI: 0.1017–0.5465), the indirect effect was (B: 0.0002, 95% CI: −0.0046–0.0051), and the total effect was (B: 0.3243).

### Stratified analyses by sex and smoking status

The significant positive associations were found in both males (HR: 1.52, 95% CI: 1.23–1.87) and females (HR: 1.68, 95% CI: 1.07–2.65) in Model 1. After adjusting for the baseline metabolic status, this association remained the same in males (HR: 1.32, 95% CI: 1.06–1.64), but the statistical significance weakened among females (HR: 1.38, 95% CI: 0.86–2.23) (interaction p = 0.69) (Table 2).

The smoking status did not seem to modify the association of the tendency to overeat with prediabetes incidence (HRs were 1.36, 1.45, and 1.21 for never, past, and current smokers, respectively, interaction p = 0.50) (Supplementary Table 1).

### Sensitivity analysis

Censoring participants who developed diabetes mellitus during follow-up did not change the association between the tendency to overeat and the incidence of prediabetes (Supplementary Table 2). Additionally, excluding participants who developed diabetes mellitus during follow-up did not alter the association between the tendency to overeat and the incidence of prediabetes (Supplementary Table 3).

## Discussion

This study identified a positive association between the tendency to overeat and incidence of prediabetes among young and middle-aged Japanese workers, independent of demographic, lifestyle, and metabolic factors. This association was observed in both males and females.

To the best of our knowledge, no cohort studies have directly explored the association between the tendency to overeat and the incidence of prediabetes in a worksite-based study. A cross-sectional study of women reported that one in five engaged in weekly overeating [14], and the Framingham Heart Study, which examined 3,551 individuals cross-sectionally [15], found that overeating was independently associated with elevated HbA1c levels, higher FBG, increased insulin resistance, independent of BMI. This highlighted that overeating may play a role in metabolic dysfunction, even in individuals without obesity. Similarly, a large cross-sectional study of Japanese men reported that eating to satiety was associated with adverse lipid profiles, including elevated TG and low-density lipoproteins cholesterol (LDL-C), even after adjustment for BMI, suggesting that overeating may influence metabolic risk independent of overall adiposity, potentially through mechanisms such as insulin resistance [17]. Moreover, just three days of overeating impaired insulin suppression of endogenous glucose production, inducing hepatic insulin resistance [18]. Additionally, seven days of overeating resulted in a 50% reduction in insulin-stimulated glucose uptake, causing insulin resistance in both muscles and adipose tissues [19]. Moreover, chronic overeating has been shown to induce insulin hypersecretion, leading to elevated insulin levels in normoglycemic, normal-weight individuals [11, 12]. These results suggested that overeating might be an important factor in relation to glycemic dysregulations.

In the current study, we explored pathway through which the tendency to overeat may increase the risk of prediabetes by conducting a mediation analysis. Differences in dietary behaviors, particularly a tendency to overeat, may contribute to an increase in BMI overtime [20, 21]. Individuals with a tendency to overeat might be more likely to consume food in response to its palatability rather than physiological energy needs, thereby promoting repeated excess energy intake [22]. As longitudinal changes in BMI could be a potential explanation for future risk of prediabetes and diabetes [23, 24], we hypothesized that tendency to overeat could increase the risk of prediabetes by influence of changes in BMI [25]. However, our mediation association suggested that longitudinal changes in BMI may not mediate the association between tendency to overeat and incident prediabetes. In the current study, the most plausible explanation would be that baseline BMI has already been the consequence of the participant’s overeating tendency before baseline. As a result, the effect of subsequent BMI changes during follow-up may have been limited after accounting for baseline BMI. Consistence with this finding, previous longitudinal study reported that longitudinal changes in BMI did not substantially alter the association between overeating and glycemic dysregulation [7, 26]. Nevertheless, other biological mechanisms have been proposed to explain the association between overeating and impaired glucose metabolism, such as hyperinsulinemia [27], oxidative stress [19], activation of sympathetic nervous system [28], endoplasmic reticulum stress caused by overeating behavior [29]. However, these mechanisms remained speculative and further research is needed to clarify their interactions.

Previous studies reported that there is an association between the former smokers and overeating [30], which has been linked to subsequent weight gain and an increased risk for diabetes mellitus [31]. These findings imply that the tendency to overeat could be particularly important in former smokers [32]. However, our study found no significant interaction between smoking status and the tendency to overeat in prediabetes incidence (Supplementary Table 1). This might be because of the differences in methodology of the studies, lifestyle and occupational behaviors across populations.

We acknowledge several limitations in the current study. First, the tendency to overeat was measured using a single non-validated question and it is not validated against established clinical instruments such as the Screen for Disorder Eating (SDE) or the Eating Disorder Examination Questionnaire (EDE-Q). Therefore, this measure may not fully capture the different dimensions of eating behaviors, and participants may have interpreted the concept of overeating differently. Consequently, the observed associations should be interpreted with caution. Nevertheless, similar single-item measures have been employed in previous longitudinal studies to identify overeating tendencies [7]. Second, during overeating episodes, certain food choices may contribute to prediabetes [11, 33]. Although we adjusted for total energy intake, energy-adjusted carbohydrates and fat intake per day, our results remained unchanged suggesting this association may be driven by the behavioral aspect of overeating. Third, we relied solely on FBG measurement and diabetes medication to identify prediabetes due to unavailability of HbA1c measurements for all the participants [34]. However, a longitudinal study conducted among the Japanese population reported while HbA1c identified fewer individuals with prediabetes compared to FBG, both measures had a similar predictive value for progression to diabetes [3]. Therefore, the use of FBG in our study could be considered a valid and reliable approach for identifying prediabetes. Fourth, while both men and women showed a protective association between the tendency to overeat and prediabetes incidence, this association was attenuated among female in the model 2. This might be because of the small number of women increasing a chance of low statistical power. Fifth, the exposure variable was assessed only at baseline, and the mediation analysis did not account for time-varying covariates. Nevertheless, given the observational study design and the absence of a formal causal mediation analysis accounting for time-dependent confounding, these findings should be interpreted cautiously. However, to partly address this limitation, we conducted supplementary time-dependent Cox-regression analyses updating age, alcohol consumption, smoking status, sleeping hours, breakfast habit, medications for hypertension and hyperlipidemia, BMI, and TG during the follow-up (Supplementary method 1). The positive association between baseline tendency to overeat and incidence of prediabetes remained generally consistent with the primary analysis in both total sample and the sex stratified analysis (Supplementary Table 4). In addition, we examined the attenuation of the association between the tendency to overeat and incidence of prediabetes after adjustment for longitudinal changes of BMI within the time-dependent Cox-regression framework (Supplementary Method 2). The results showed minimal attenuation (0.55%) after adjustment for longitudinal changes of BMI (Supplementary Table 5). However, exposure was measured only at baseline, therefore these findings should be interpreted cautiously. Sixth, given the relatively homogeneous nature of the current study population in terms of their socioeconomic status, which could increase the internal validity of the present study, may not be able to generalize to other populations. Seventh, unmeasured or residual confounding may have influenced the results, despite our effort to adjust to a wide range of potential covariates. In particular, unmeasured overeating tendency or metabolic status before baseline may have influenced both the self-reported tendency to overeat at baseline and subsequent risk of prediabetes. The more favorable metabolic profile observed at the baseline among participants who reported no tendency to overeat may reflect such prior eating behaviors or metabolic status. Although we included measured baseline metabolic factors in Model 2, such as BMI, and TG, these factors may not fully capture prior overeating tendency or metabolic status. Therefore, residual confounding due to unmeasured factors cannot be completely excluded. Finally, although participants with prevalent prediabetes and diabetes were excluded at baseline, reverse causation remains possible. Individuals with early metabolic changes that had not yet met the diagnostic criteria for prediabetes may have modified their eating behaviors, which could have influenced their self-reported tendency to overeat at baseline. Consequently, the possibility of reverse causation cannot be entirely ruled out.

In terms of implementation, the national health check-up lifestyle questionnaire does not include a specific question on identifying the tendency to overeat and not tending to overeat at the nationwide level. Therefore, a targeted screening for this could enhance risk assessment and contribute to more effective prevention strategies, potentially reducing incidence of prediabetes.

## Conclusion

The present study suggested that the tendency to overeat was positively associated with incidence of prediabetes in young and middle-aged Japanese workers, independent of socio-demographic factors, lifestyle factors, baseline metabolic status and metabolic state changes. This association was similar among both males and females.

## Abbreviations

BMI: Body Mass Index
WC: Waist Circumference
FBG: Fasting Blood Glucose
TG: Triglycerides
LDL-C: Low density Lipoprotein Cholesterol
HbA1c: Glycated Hemoglobin A1c
DAG: Directed Acyclic Graph

## Supplementary information

**Additional file 1:** **Supplementary Figure 1.** Directed Acyclic Graph showing relationships between the tendency to overeat, confounders, metabolic status, and prediabetes incidence. **Supplementary Table 1.** Tendency to overeat and prediabetes incidence by smoking status: Cox regression. **Supplementary Table 2.** Tendency to overeat and prediabetes incidence, with censoring of diabetes mellitus cases: Cox regression. **Supplementary Table 3.** Tendency to overeat and prediabetes incidence, excluding diabetes mellitus cases: Cox regression. **Supplementary Method 1.** Tendency to overeat and prediabetes: Time-dependent Cox regression. **Supplementary Table 4.** Tendency to overeat and prediabetes: Time-dependent Cox regression. **Supplementary Method 2.** Attenuation analysis: Time-dependent Cox-regression. **Supplementary Table 5.** Attenuation analysis: Time-dependent Cox-regression. **Supplementary Figure 2.** Directed Acyclic Graph (DAG) showing alternative causal framework; relationship between tendency to overeat and incidence of prediabetes. **Supplementary Method 3.** Cox-regression analysis based on an alternative causal framework. **Supplementary Table 6.** Tendency to overeat and prediabetes: Cox regression analysis under an alternative causal framework: Aichi Workers’ Cohort Study, n = 2886.

## Declarations

### Data availability

The datasets generated and/or analyzed during the current study are not publicly available due to ethical concerns but are available from the corresponding author on reasonable request after appropriate procedures including approval from the institutional ethics review committee.
