# Supplementary material for: Association between tendency to overeat and the incidence of prediabetes: Aichi Workers’ Cohort Study
Source: Environ Health Prev Med. 2026 Sep 10;31:59. doi: 10.1265/ehpm.26-00084 (PMC13585473; doi:10.1265/ehpm.26-00084)
Supplement: Supplementary file 1 [file ehpm-31-059-s001.docx]

**Supplementary Figure 1. Directed Acyclic Graph showing relationships between tendency to overeat, confounders, metabolic status, and prediabetes incidence**.

Note: This graph illustrates the hypothesized relationship between tendency to overeat, confounders, baseline metabolic status, metabolic state change and prediabetes incidence. Baseline metabolic state indicates baseline body mass index (BMI), and triglyceride (TG), fasting blood glucose (FBG). Metabolic state change indicates longitudinal changes of BMI. Confounders include age, sex, job type, physical activity, alcohol consumption, smoking status, sleeping hours, breakfast habit, mental stress and medications for hypertension and hyperlipidemia. The indirect effect of the tendency to overeat (independent variable; X) on the incidence of prediabetes (dependent variable; Y) through the metabolic state changes (mediator; M). a pathway is the effect of X on M. b pathway is effect of M on Y. The mediated or indirect effect is a*b pathways. c` pathway is the direct effect of X on Y. The total effect (c pathway) of the tendency to overeat (independent variable; X) on the incidence of prediabetes (dependent variable; Y).

| **Supplementary Table 1. Tendency to overeat and prediabetes incidence by smoking status: Cox regression.** | | |
| --- | --- | --- |
|  | Tendency to overeat | |
|  | No | Yes |
| **Never- smokers** (n/N) | 62/ 443 | 147/ 720 |
| Crude incidence rate (/ 1000 person-years) | 32.5 | 48.9 |
| Model 2 | 1 (Ref) | 1.36 (0.99-1.86) |
| **Past- smokers** (n/N) | 34/ 189 | 106/ 380 |
| Crude incidence rate (/ 1000 person-years) | 44.7 | 70.7 |
| Model 2 | 1 (Ref) | 1.45 (0.95-2.20) |
| **Current- smokers** (n/N) | 42/158 | 53/ 196 |
| Crude incidence rate (/ 1000 person-years) | 68.9 | 65.3 |
| Model 2 | 1 (Ref) | 1.21 (0.74-1.99) |
| n indicates the number of incident prediabetes; N, the number of participants; Ref, reference  Note: Analysis restricted to males from the Aichi Workers’ Cohort Study (n = 2086).  Model 2 includes age, job type, physical activity, alcohol consumption, sleeping hours, breakfast habit, mental stress, medications for hypertension and hyperlipidemia, body mass index, triglycerides.  P for interaction between tendency to overeat categories and smoking status categories is 0.49. | | |

| **Supplementary Table 2. Tendency to overeat and prediabetes incidence, with censoring of diabetes mellitus cases: Cox regression.** | | |
| --- | --- | --- |
|  | Tendency to overeat | |
|  | No | Yes |
| **Total participants** (n/N) | 161/ 1,106 | 362/ 1,780 |
| Crude incidence rate (/ 1000 person-years) | 34.5 | 48.8 |
| Model 1 | 1 (Ref) | 1.54 (1.27-1.87) |
| Model 2 | 1 (Ref) | 1.32 (1.08-1.61) |
| **Male participants** (n/N) | 133/ 790 | 297/ 1,296 |
| Crude incidence rate (/ 1000 person-years) | 40.6 | 55.8 |
| Model 1 | 1 (Ref) | 1.53 (1.23-1.89) |
| Model 2 | 1 (Ref) | 1.32 (1.06-1.65) |
| **Female participants** (n/N) | 28/ 316 | 65/ 484 |
| Crude incidence rate (/ 1000 person-years) | 20.2 | 31.0 |
| Model 1 | 1 (Ref) | 1.59 (1.01-2.52) |
| Model 2 | 1 (Ref) | 1.30 (0.80-2.11) |
| n indicates the number of incident prediabetes; N, the number of participants; Ref, reference  Note: Analysis conducted in the Aichi Workers’ Cohort Study with a total sample size of n = 2886. Participants who developed diabetes mellitus were censored at the time of diabetes mellitus diagnosis during follow-up.  Model 1 includes age, sex, job type, physical activity, alcohol consumption, smoking status, sleeping hours, breakfast habit, mental stress and medications for hypertension and hyperlipidemia. In the sex-stratified analyses, sex was not included as a covariate. Model 2 includes Model 1 and body mass index, triglycerides. | | |

| **Supplementary Table 3. Tendency to overeat and prediabetes incidence, excluding diabetes mellitus cases: Cox regression.** | | |
| --- | --- | --- |
|  | Tendency to overeat | |
|  | No | Yes |
| **Total participants** (n/N) | 160/ 1100 | 355/ 1761 |
| Crude incidence rate (/ 1000 person-years) | 34.4 | 48.2 |
| Model 1 | 1 (Ref) | 1.53 (1.26-1.85) |
| Model 2 | 1 (Ref) | 1.32 (1.08-1.61) |
| **Male participants** (n/N) | 132/ 784 | 290/ 1,281 |
| Crude incidence rate (/ 1000 person-years) | 40.4 | 54.9 |
| Model 1 | 1 (Ref) | 1.51 (1.22-1.86) |
| Model 2 | 1 (Ref) | 1.32 (1.06-1.66) |
| **Female participants** (n/N) | 28/ 316 | 65/ 480 |
| Crude incidence rate (/ 1000 person-years) | 20.2 | 31.2 |
| Model 1 | 1 (Ref) | 1.60 (1.01-2.53) |
| Model 2 | 1 (Ref) | 1.31 (0.80-2.12) |
| n indicates the number of incident prediabetes; N, the number of participants; Ref, reference  Note: Analysis conducted in the Aichi Workers’ Cohort Study with a total sample size of n = 2861. Participants who developed diabetes mellitus during follow-up were excluded from the analysis.  Model 1 includes age, sex, job type, physical activity, alcohol consumption, smoking status, sleeping hours, breakfast habit, mental stress and medications for hypertension and hyperlipidemia. In the sex-stratified analyses, sex was not included as a covariate. Model 2 includes Model 1 and body mass index, triglycerides. | | |

**Supplementary Method 1**

Time- dependent Cox- regression hazard analysis was conducted in R version 4.5.1 (R foundation for statistical computing, Vienna, Austria) using the *survival* package. Data management and preparation were performed using the *dplyr* package. A counting- process data structure was created with one record for each participant during each follow-up interval, defined by start and stop. Baseline covariates remained fixed, whereas repeatedly measured covariates were updated at each follow-up interval. Updated covariates are as follows; age, alcohol consumption, smoking status, sleeping hours, breakfast habit, medications for hypertension and hyperlipidemia., BMI, and TG during the follow-up When follow-up information for time-varying covariates was missing, the last observed value was carried forward.

Two time-dependent Cox- regression models were constructed. In model 1, tendency to overeating was examined in relation to incident of prediabetes with adjustment for age, sex, job type, physical activity, alcohol consumption, smoking status, sleeping hours, breakfast habit, mental stress and medications for hypertension and hyperlipidemia. Time-varying covariates were updated during follow-up where repeated measurements were available. Model 2 was additionally adjusted for time-updated BMI and TG levels.

| **Supplementary Table 4. Tendency to overeat and prediabetes: Time- dependent Cox regression, Aichi Workers' Cohort Study, n=2886.** | | |
| --- | --- | --- |
|  | Tendency to overeat | |
|  | No | Yes |
| **Total participants** (541/ 2886) |  |  |
| Model 1 | 1 (Ref) | 1.57 (1.30- 1.89) |
| Model 2 | 1 (Ref) | 1.32 (1.08-1.61) |
| **Male participants** (444/ 2086) |  |  |
| Model 1 | 1 (Ref) | 1.54 (1.25-1.90) |
| Model 2 | 1 (Ref) | 1.30 (1.05-1.62) |
| **Female participants** (97/ 800) |  |  |
| Model 1 | 1 (Ref) | 1.73 (1.10-2.71) |
| Model 2 | 1 (Ref) | 1.38 (0.86-2.23) |
| n indicates the number of incident prediabetes; N, the number of participants; Ref, reference  Note: Model 1 included time-updated age, alcohol consumption, smoking status, sleeping hours, breakfast habit, medications for hypertension and hyperlipidemia, with baseline METs, total energy intake, job type, PSS-4 score, sex and tendency to overeat. In the sex-stratified analyses, sex was not included as a covariate. Model 2 additionally adjusted for time-updated TG, BMI.  P for interaction between tendency to overeat categories and sex categories are 0.60 | | |

Supplementary Method 2

A separate attenuation analysis was conducted to evaluate whether BMI change explained the association between tendency to overeating and incidence of prediabetes when using a time- dependent Cox- regression framework. This analysis was performed separately from the time- dependent cox- regression analysis, model 2. To maintain consistency with the original mediation framework in the manuscript, baseline BMI was included as a fixed covariate, while BMI changes were examined as the potential mediator.

The reference model included time updated age, alcohol consumption, smoking status, sleeping hours, breakfast habit, medications for hypertension and hyperlipidemia, TG, with baseline BMI, METs, total energy intake, job type, PSS-4 score, sex and baseline tendency to overeat. A second model additionally included longitudinal changes of BMI. The percentage attenuation of the overeating coefficient was calculated as:

*Percent attenuation* = $\frac{\beta reference- \beta BMI change model}{\beta reference}$×100

β reference= Regression coefficient for tendency to overeating from the reference time- dependent Cox- regression hazard model.

β BMI change model= Regression coefficient for tendency to overeat after adding longitudinal changes of BMI to reference model.

| **Supplementary Table 5. Attenuation Analysis: Time Dependent Cox- regression analysis.** | | |
| --- | --- | --- |
| Model | HRs (95% CIs) | β Attenuation (%) |
| Reference model | 1.33 (1.09-1.63) | 0.546 |
| Reference model + BMI changes | 1.33 (1.09- 1.62) |  |
| HR, hazard ratio; CI, confidence interval  This table represents attenuation analysis of the association between the tendency to overeat and incidence prediabetes after adjusting for BMI change within the time-dependent Cox-regression hazard model.  Note: The reference model included time updated age, alcohol consumption, smoking status, sleeping hours, breakfast habit, medications for hypertension and hyperlipidemia, TG, with baseline BMI, METs, total energy intake, job type, PSS-4 score, sex and baseline tendency to overeat. A second model additionally included longitudinal changes of BMI. | | |

**Supplementary Figure 2. Directed Acyclic Graph (DAG) showing alternative causal framework; relationship between tendency to overeat and incidence of prediabetes.**

Note: Alternative directed acyclic graph (DAG) illustrates the hypothesized causal relationships between tendency to overeat and incident prediabetes under an alternative causal framework. Age, sex, job type, physical activity, alcohol consumption, smoking status, sleeping hours, breakfast habit, and mental stress were considered confounding variables. Baseline BMI, baseline triglycerides, medications for hypertension and hyperlipidemia, and longitudinal BMI change were assumed to lie on the hypothesized causal pathway between tendency to overeat and incident prediabetes.

**Supplementary Method 3**

A Cox proportional hazards regression model was used to estimate hazard ratios (HRs) and 95% confidence intervals (CIs) for incident prediabetes. Based on the alternative DAG (Supplementary Figure 2), one multivariable model was constructed adjusting for age, sex, job type, physical activity, alcohol consumption, smoking status, sleeping hours, breakfast habit, and mental stress. Baseline BMI, baseline TG, and medications for hypertension and hyperlipidemia were not included in the adjustment model. Analyses were conducted for the total study population and separately for males and females. Effect modification by sex was evaluated using an interaction term between tendency to overeat and sex.

| **Supplementary Table 6. Tendency to overeat and prediabetes: Cox regression analysis under an alternative causal framework: Aichi Workers' Cohort Study, n=2886.** | | |
| --- | --- | --- |
|  | Tendency to overeat | |
|  | No | Yes |
| **Total participants** (n/N) | 166/1,106 | 375/1,780 |
| Crude incidence rate (/ 1000 person-years) | 35.6 | 50.6 |
| Multivariable model | 1 (Ref) | 1.57 (1.30-1.89) |
| **Male participants** (n/N) | 138/ 790 | 306/ 1,296 |
| Crude incidence rate (/ 1000 person-years) | 42.1 | 57.5 |
| Multivariable model | 1 (Ref) | 1.54 (1.26-1.90) |
| **Female participants** (n/N) | 28/ 316 | 69/484 |
| Crude incidence rate (/ 1000 person-years) | 20.2 | 32.6 |
| Multivariable model | 1 (Ref) | 1.67 (1.06-2.61) |
| n indicates the number of incident prediabetes; N, the number of participants; Ref, reference  Note: Multivariable model includes age, sex, job type, physical activity, alcohol consumption, smoking status, sleeping hours, breakfast habit, and mental stress. In the sex-stratified analyses, sex was omitted from the adjustment.  P for interaction between tendency to overeat categories and sex categories are 0.71 | | |
